# Formulation Development of a Food-Graded Curcumin-Loaded Medium Chain Triglycerides-Encapsulated Kappa Carrageenan (CUR-MCT-KC) Gel Bead Based Oral Delivery Formulation

**DOI:** 10.3390/ma14112783

**Published:** 2021-05-24

**Authors:** Kei-Xian Tan, Ling-Ling Evelyn Ng, Say Chye Joachim Loo

**Affiliations:** 1 School of Materials Science and Engineering, Nanyang Technological University, Singapore 639798, Singapore; evelynisabellia@gmail.com; 2 Esco Aster, Block 71, Ayer Rajah Crescent, Singapore 139951, Singapore; 3 Singapore Centre for Environmental Life Sciences Engineering, Nanyang Technological University, 60 Nanyang Drive, Singapore 637551, Singapore; 4 Harvard T.H. Chan School of Public Health, Harvard University, 677 Huntington Ave, Boston, MA 02115, USA

**Keywords:** drug delivery system, biodegradable, biomaterials, pharmaceutical, nutraceuticals

## Abstract

In recent years, curcumin has been a major research endeavor in food and biopharmaceutical industries owing to its miscellaneous health benefits. There is an increasing amount of research ongoing in the development of an ideal curcumin delivery system to resolve its limitations and further enhance its solubility, bioavailability and bioactivity. The emergence of food-graded materials and natural polymers has elicited new research interests into enhanced pharmaceutical delivery due to their unique properties as delivery carriers. The current study is to develop a natural and food-graded drug carrier with food-derived MCT oil and a seaweed-extracted polymer called k-carrageenan for oral delivery of curcumin with improved solubility, high gastric resistance, and high encapsulation of curcumin. The application of k-carrageenan as a structuring agent that gelatinizes o/w emulsion is rarely reported and there is so far no MCT-KC system established for the delivery of hydrophobic/lipophilic molecules. This article reports the synthesis and a series of in vitro bio-physicochemical studies to examine the performance of CUR-MCT-KC as an oral delivery system. The solubility of CUR was increased significantly using MCT with a good encapsulation efficiency of 73.98 ± 1.57% and a loading capacity of 1.32 ± 0.03 mg CUR/mL MCT. CUR was successfully loaded in MCT-KC, which was confirmed using FTIR and SEM with good storage and thermal stability. Dissolution study indicated that the solubility of CUR was enhanced two-fold using heated MCT oil as compared to naked or unformulated CUR. In vitro release study revealed that encapsulated CUR was protected from premature burst under simulated gastric environment and released drastically in simulated intestinal condition. The CUR release was active at intestinal pH with the cumulative release of >90% CUR after 5 h incubation, which is the desired outcome for CUR absorption under human intestinal conditions. A similar release profile was also obtained when CUR was replaced with beta-carotene molecules. Hence, the reported findings demonstrate the potencies of MCT-KC as a promising delivery carrier for hydrophobic candidates such as CUR.

## 1. Introduction

Curcumin (CUR) is a biologically active, non-polar, and naturally occurring polyphenolic compound from turmeric that possesses a variety of health benefits [1]. It is the most active curcuminoid present in the flowering plant called *Curcuma longa* and is responsible for the yellow pigmentation of turmeric. Given its antimicrobial, antioxidant, antitumor, anti-inflammatory, and immunostimulatory properties, it has become one of the most widely studied food-derived compound [2]. Because of this, CUR is frequently used as a lipophilic compound in functional food.

Despite its health benefits, its usage is limited by its poor water solubility, stability, and bioavailability [3,4]. CUR undergoes rapid degradation, molecular fragmentation, and metabolic inactivation at a physiological pH. In addition, both alkaline and a neutral CUR solution can be degraded at room temperature and the degradation is more rapid at higher temperatures such as human body temperature 37 °C. CUR is also photochemical-sensitive whereby it undergoes degradation upon sunlight exposure. Because of its low water solubility (~11 ng/mL), it usually ends up in feces [5]. Pharmacokinetic data shows that orally administered CUR in rodents and humans is low, including 0.22 μg/mL from 1 g/kg CUR in mouse [6] and 0.051 μg/mL from 12 g CUR in humans [7]. To improve bioavailability, there is therefore a need to encapsulate CUR by enhancing its solubility.

One of the most suitable modes of delivery for CUR is through emulsions. Both CUR nano- and micro-emulsions have been extensively applied in drug delivery, the cosmetic sector, and food industry, for effective delivery of hydrophobic molecules [8]. However, the use of kappa (κ-) carrageenan (KC) as a prime bulk phase structuring agent with gelation of CUR encapsulated in oil-in-water (O/W) emulsion is scarcely studied.

Medium-chain triglycerides (MCT) and KC employed in this study are generally recognized as safe (GRAS), non-toxic, edible, biodegradable, biocompatible, and biologically safe. These are interesting features for biopharmaceutical and food industries, owing to the contemporary direction of green consumerism. CUR in an edible medium such as MCT oil and KC, can be directly used in food or pharmaceutical ingredients, without the requirement of eliminating the extraction medium. MCT are triglycerides with two or three fatty acids and a short chain length of 6–12 carbon. It can be extracted from food sources such as coconut oil and palm kernel oil [9]. Moreover, the use of MCT in food products are approved with GRAS status by US FDA in 1994, which expands their applications in foods, cosmetics, nutrition, and drugs [10]. They are gaining increasing attention due to their unique bioactivities, such as lowering the cholesterol level, slowing weight gain, and increasing ketone production [11]. MCT as a carrier lipid has also been reported to significantly increase the CUR bioaccessibility [9,12,13].

KC, on the other hand, is a linear, anionic, hydrophilic, and sulphated polygalactan naturally derived from red seaweeds of the class *Rhodophyceae* [12]. It is made up of repeating D-galactose residues with one negative charge per disaccharide unit [13]. KC comprises D-galactose-4-sulfate and 3,6-anhydro-D-galactose. Due to its distinctive characteristics, carrageenan is extensively used in various biomedical applications and therapeutic treatments, as reported in numerous studies [14,15,16,17]. It has been exploited as the safe food additive for decades in meat, milk, and yogurt. This suggests the biocompatibility, functionality, and potencies of carrageenan as an ideal excipient for pharmaceutical delivery. Food-graded carrageenan has been deemed safe for its use in infant formulation (300 mg/L) by the Joint FAO/WHO Expert Committee on Food Additives (JECFA) [18] and it has been classified as non-carcinogenic by the International Agency for Research on Cancer (IARC) [19,20]. Furthermore, carrageenan is included in the British Pharmacopoeia 2012, US Pharmacopeia 35-National Formulary 30 S1 and European Pharmacopoeia 7.0, inferring its promising potential in the development of pharmaceutical formulations, taking into account its biological characteristics such as anti-herpes, anti-HIV, anticoagulant, anti-tumor, and immunomodulatory activities [21].

In the present study, a modified O/W single emulsion technique is reported to prepare the CUR-MCT-KC with better properties by using MCT as a suitable oil phase for CUR encapsulation and KC as the aqueous phase that gelatinizes CUR-MCT emulsion into gel beads. Emulsion is a kinetically stable, non-equilibrated, and colloidal system comprising two or more immiscible liquids. There are various studies that have reported the use of either MCT or KC natural compounds for the encapsulation, delivery, and stabilization of CUR [1,9,22,23,24,25,26]. However, to the best of our knowledge, the use of both MCT oil and KC polysaccharides as an effective oral delivery carrier for CUR and their synergetic effects has never been reported before.

This research work, the first of its kind, was able to fabricate CUR-MCT-KC gel beads and evaluate the efficiency of MCT oil and KC polysaccharides for CUR emulsification and high gastric resistance. The aim of this study was to design and develop a natural, food-graded oral delivery system for the delivery of CUR and examine the synergetic effects of MCT-KC for better encapsulation, solubility, and release of CUR. The in vitro experiments were carried out on the hypothesis that a MCT-KC nature-derived formulation can stabilize, encapsulate, and deliver CUR effectively via the gastrointestinal tract (GIT) when administrated orally. The differences between CUR-KC, CUR-MCT, and CUR-MCT-KC were also examined and compared to demonstrate the potential of CUR-MCT-KC formulation. This work can therefore provide useful in vitro information for the development of an oral delivery system of other nutraceuticals, with poor solubility to further enhance their loading capacity and practical applications.

## 2. Materials and Methods

### 2.1. Materials

Curcumin (*Curcuma longa* (Turmeric), powder), KC (sulfated plant polysaccharide), phosphate-buffered saline (PBS) (10× concentrate, pH 7.4), potassium chloride (KCl) (molecular weight 74.55, ≥99%), acetonitrile (ACN) (HPLC grade, ≥99%), dichloromethane (DCM), bile salts (Dehydrated, purified fresh bile), Tween 20 (polysorbate 20), and Span 20 (sorbitan monolaurate) were purchased from Sigma Aldrich, Singapore. MCT oil (Neobee 1053) was purchased from Stepan company, Northfield, IL, USA. Deionized (DI) water was used in all the experiments.

### 2.2. Preparation of Curcumin-Loaded Medium Chain Triglycerides-Encapsulated Kappa Carrageenan (CUR-MCT-KC) Bead Formulation

CUR encapsulation was carried out using a single O/W emulsion technique. This method is based on the emulsification of CUR-MCT organic solution into a water phase. Basically, the CUR molecules were first dissolved in MCT oil as an oil phase and Span 20 as the surfactant. The organic phase was then further emulsified in the continuous/aqueous phase made up of KC. Emulsification was conducted via homogenization under high-shear force to reduce the size of the CUR-MCT-KC emulsion droplet and thus, final particle size.

Initially, 5 mg CUR was dissolved in 1 mL MCT oil and Span 20 (10%) by stirring the mixture at 60 °C with gentle shaking at 150 rpm for 5 min or until an orange solution was formed; 60 °C was adopted because it is a temperature that can be replicated in industrial conditions. The temperature was increased to 120 °C if the CUR was not dissolved completely; 2% *w*/*v* KC solution was prepared in distilled water by adding 2 g KC into 100 mL distilled water with gentle shaking at 350 rpm, 60−70 °C for 5 min or until the complete dissolution of KC to obtain a clear and homogenous KC solution. To fabricate the O/W emulsion, the KC solution was mixed with the MCT oils containing CUR (0.5% *w*/*v* CUR-MCT solution) via homogenization at 80 °C with 13,000 rpm for 5 min.

Finally, the CUR-MCT-KC bead production was conducted via a crosslinking process using KCl as crosslinkers. The above-prepared CUR-MCT-KC solution was dropped through a 1 mL syringe with a needle into a beaker containing a 5% KCl aqueous phase. Beads were formed and collected in the aqueous phase, resulting in spherical CUR-MCT-KC beads, as displayed in Appendix A (Figure A1 and Figure A2). Next, the beads were collected and dried using filter papers prior to air-drying them in a light-protected container at room temperature for the beads to be hardened.

Similar procedures were carried out for the fabrication of CUR-MCT-KC beads containing a higher concentration of MCT (CUR-MCT-KC_2) using 5 mL MCT to dissolve 5 mg CUR (0.1% *w*/*v* CUR-MCT solution) before mixing with 0.4% *w*/*v* KC solution. On the other hand, beta-carotene-MCT-KC (BC-MCT-KC) beads were also fabricated using the same techniques and procedures by replacing CUR with BC molecules.

### 2.3. Biophysical Characterization of CUR-MCT-KC Formulation

#### 2.3.1. Scanning Electron Microscopy (SEM)

SEM JSM-6360 (JEOL, Ltd., Tokyo, Japan,) was used to visualize the size and surface morphology of the CUR-MCT-KC bead. SEM is a technology that uses the focused electron beams and different signals to obtain the sample image. The samples were mounted onto carbon tape prior to the sputter coating with gold for 45 s. Each sample was then imaged at different magnification of 35×, 50×, 150×, 550×, and 1000× with a 5 kV voltage and 20 mm working distance.

#### 2.3.2. UV-Visible Spectrophotometric Analysis

The presence of CUR can be identified by the maximum absorption peak that can be determined using UV-visible spectrophotometry at the wavelength of 425 nm. This peak is mainly due to the π–π type excitation of the CUR aromatic system [27]. The properties of the absorbance intensity changes at 425 nm were used to examine the solubility, dissolution, release, and stability of the fabricated CUR-MCT-KC formulation, using Infinite^®^ M200 (Tecan Group Ltd, Menendorf, Switzerland). To ensure the measurement accuracy, 300 µL volume was standardized for every analysis made. Moreover, the wavelength used for BC measurements was 448 nm.

#### 2.3.3. Thermogravimetric Analysis (TGA)

The thermal stability and behavior of CUR-MCT-KC beads was investigated using TGA (TA instrument Q500, TA Instruments, A Division of Waters Pacific Pte. Ltd., Singapore) to evaluate the change in mass at a constant heating rate in an inert environment. This is essential to determine the thermal stability and indicate the potential molecular rearrangement within the matrices of the CUR-MCT-KC complexation in terms of exotherm. The TGA instrument was purged with nitrogen at 20 °C/min and equilibrated at 25 °C prior to the analysis. Approximately 13 mg CUR-MCT-KC was placed in an aluminum pan and analyzed from 30 to 600 °C with 10 °C per min of constant heating rate and 25 mL/min of nitrogen gas. STARe software was applied for the result analysis.

### 2.4. Chemical Analysis of CUR-MCT-KC Formulation

In order to indicate the formation of CUR-MCT-KC and the presence of functional groups in CUR-MCT-KC, CUR, MCT oil, and CUR-MCT, prepared samples were characterized using the Fourier Transform Infrared Spectroscopy (FTIR) (Perkin Elmer Frontier, Waltham, MA, USA). Potassium bromide (KBr) was utilized as the background pellet and each sample (CUR, MCT, CUR-MCT, CUR-MCT-KC) was ground with the KBr in the ratio of 1:4 to form pellet using the hydraulic press. Spectra were measured in the range of 400–4000 cm^−1^ at a 4 cm^−1^ resolution. Sixteen scans for each sample were conducted to lower the sound-to-noise ratio.

### 2.5. Determination of CUR Encapsulation Efficiency (EE)

The quantity of CUR encapsulated within MCT-KC was evaluated from the difference between the initial amount of CUR added in the formulation and the amount of free CUR measured in the medium upon the breakdown of MCT-KC. Next, 10 mg CUR-MCT-KC bead was added into 1 mL distilled water and heated at 60 °C for 5 min or until a clear and homogenous solution was obtained and 1 mL ACN was added to the mixture prior to centrifugation at 13,000 rpm for 3 min; 300 µL supernatant was then collected for UV-spectrophotometry analysis at 425 nm. All absorbance readings were measured in triplicate and averaged. The CUR concentration was determined using the standard curve of CUR in ACN. The encapsulation efficiency (EE) was calculated from the following equation:

(1) $$\mathrm{Encapsulation}\;\mathrm{efficiency}\;(\%)=\frac{Amount\;of\;encapsulated\;CUR}{Total\;initial\;amount\;of\;CUR\;added\;in\;CUR-MCT-KC\;beads}\;\times \;100\%$$

### 2.6. Measurement of CUR Solubility in MCT Oil

In 1 mL MCT oils, an excess amount of CUR, 10 mg was added and heated to 60 °C under stirring to ensure complete dissolution of CUR in MCT. The temperature was increased to ~120 °C if the CUR was not dissolved completely. After cooling to 25 °C, the mixture was centrifuged at 13,000 rpm for 5 min to collect the supernatant (CUR-saturated MCT oil) prior to the UV-spectrophotometry analysis at 425 nm. The following equation was used to calculate the loading capacity:

(2) $$\mathrm{Loading}\;\mathrm{capacity}\;(\mathrm{mg}/\mathrm{mL})=\frac{Amount\;of\;encapsulated\;CUR}{Total\;amount\;of\;MCT\;applied}$$

#### The Effect of Heat on the Solubility of CUR in MCT Oil

The effect of heat on the CUR solubility in MCT oil was studied over a temperature range of 37–100 °C using a water bath. 1 mg/mL CUR was prepared using MCT and heated from 37 to 100 °C. At specific intervals (37 °C, 50 °C, 60 °C, 70 °C, 80 °C, 90 °C, 100 °C), samples were collected to be examined spectrophotometrically at 425 nm, as stated in Section 2.6 above. All absorbance readings were measured in triplicate and averaged.

### 2.7. In Vitro Dissolution Study of CUR-MCT Formulation

A dissolution study is essential to understand the solubility of naked/unformulated CUR as compared to a CUR-MCT mixture in PBS buffer that mimics the human physiological environment at pH 7.4, temperature 37 °C, and 150 rpm in a shaking incubator. Two different samples were prepared: (1) 4.15 µg/mL CUR solution prepared using PBS; the saturated CUR concentration reported in PBS; (2) 1.32 ± 0.03 mg/mg CUR-MCT solution prepared using MCT; the saturated CUR concentration in MCT determined from our study. Samples were mixed with 5 mL PBS (+0.02% Tween 20) and incubated at 37 °C, 150 rpm for 1 h. At every time interval (5th, 10th, 30th, 45th, 60th min), samples were collected for centrifugation and UV-spectrophotometer measurements at 425 nm. The CUR concentration was determined using the standard curve of CUR in PBS. The percentage of CUR dissolved was calculated from the following equation:

(3) $$\mathrm{Dissolved}\;\mathrm{CUR}\;(\%)=\frac{Dissolved\;CUR\;in\;PBS\;}{Total\;initial\;amount\;of\;CUR\;added}\;\times \;100\%$$

### 2.8. In Vitro Release Study of CUR-MCT-KC Formulation

#### 2.8.1. In Vitro Release Profile at Different pH

The effect of pH on encapsulation efficiency of CUR-MCT-KC and CUR-KC was evaluated and compared via the in vitro release study at extreme acidic and alkali pHs: pH 1.2 and pH 7.4. 2 mg of the CUR-MCT-KC bead was incubated in 1 mL PBS (+0.02% Tween 20) at pH 1.2, 150 rpm, and temperature 37 °C for 2 h in a shaking incubator. At every time interval (5th, 15th, 30th, 60th, 90th, 120th min), a supernatant was collected to be measured spectrophotometrically at 425 nm to identify the amount of released CUR. Fresh PBS was added to replenish the extracted sample at every time interval; the pH of PBS was adjusted using 0.1 M sodium chloride (NaCl) or 0.1 M potassium hydroxide (NaOH) to the respective pH level. The above procedures were repeated for CUR-MCT-KC bead under the alkali condition at pH 7.4, and a release study of CUR-KC bead at both pH 1.2 and pH 7.4. The percentage of CUR released was calculated from the following equation:

(4) $$\mathrm{Released}\;\mathrm{CUR}\;(\%)=\frac{Released\;CUR\;from\;CUR-MCT-KC\;or\;CUR-KC\;beads\;}{Total\;initial\;amount\;of\;CUR\;added\;in\;CUR-MCT-KC\;or\;CUR-KC\;beads}\;\times \;100\%$$

#### 2.8.2. In Vitro Release Profile in Simulated GI Conditions

The release mechanism of encapsulated CUR under simulated GI conditions was examined by mimicking the physiological environment in the upper tract (stomach and small intestine) of human GIT. The CUR-MCT-KC gel beads (2 mg) were incubated in 1 mL simulated gastric fluid (SGF) +0.02% Tween 20 at pH 1.2, temperature 37 °C, 150 rpm in a shaking incubator for 2 h, which represents the average transition time of GI. The entire sample after 2 h of gastric digestion was then transferred to the simulated intestinal fluid (SIF) +0.3% bile salts for a subsequent incubation of 3 h. At specific time intervals (5th, 15th, 30th, 60th, 90th, 120th, 180th, 240th, 300th min), 1 mL supernatant was collected and mixed with 1 mL DCM prior to centrifugation at 13,000 rpm for 3 min. This is to separate the released CUR from the loaded beads. Free CUR is very soluble in organic solvent–DCM. The CUR-DCM supernatant was collected and vacuum-dried. The released CUR was re-dissolved in 1 mL ACN to assay spectrophotometrically at 425 nm. All absorbance readings were measured in triplicate and averaged. Fresh SGF or SIF was added to replenish the extracted sample at every time interval. The concentration of released CUR was then determined using the standard curve of CUR in ACN. The percentage of CUR released was calculated from the following equation:

(5) $$\mathrm{Released}\;\mathrm{CUR}\;(\%)=\frac{Released\;CUR\;from\;CUR-MCT-KC\;beads\;}{Total\;initial\;amount\;of\;CUR\;added\;in\;CUR-MCT-KC\;beads}\;\times \;100\%$$

The above procedures were replicated for in vitro release study of CUR-MCT-KC, CUR-MCT emulsion in simulated GI conditions, and BC-MCT-KC beads in different conditions: (1) SGF for 5 h; (2) SIF for 5 h; (3) SGF for the first 2 h and SGF for the subsequent 3 h. Chloroform was used instead of DCM in the extraction of BC due to its solubility in different organic solvents. Moreover, the wavelength used for BC measurement was 448 nm.

### 2.9. Experimental Analysis

Each block of experiment was conducted in triplicate (minimum), and the average measured values were reported as the final analytical data. Experimental data are recorded as average ± standard deviation and/or standard error.

## 3. Results and Discussion

### 3.1. Fabrication and Biophysical Characterization of CUR-MCT-KC Oral Delivery System

#### 3.1.1. CUR-MCT-KC Design Concept and Optimization

**Figure 1:**
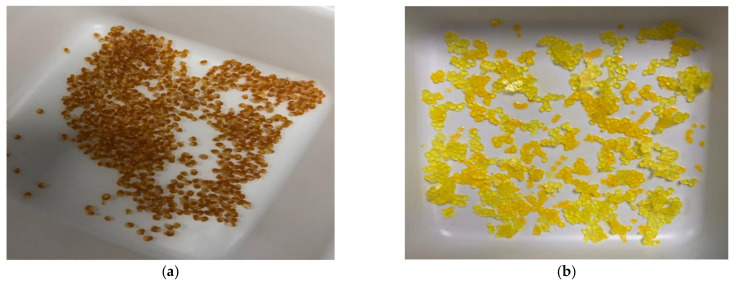
Dried and solidified CUR-MCT-KC and CUR-MCT-KC_2 beads with (**a**) 0.5% *w*/*v* CUR-MCT, (**b**) 0.1% *w*/*v* CUR-MCT before encapsulated with KC solution. Beads with higher amount of MCT were more yellowish due to a more even CUR distribution.

Based on the literature, the use of natural oils (e.g., corn oils, olive oils, black pepper oils, LCT oils, MCT oils) and natural polymers such as KC, as a delivery carrier for hydrophobic CUR molecules [1,22,23,26,28,29,30,31,32,33,34,35,36], has been reported extensively. However, because of the gastric susceptibility of CUR, many reported formulations encounter the challenge of high gastric digestion. In this study, the aim was to report on a high encapsulation efficiency, a gastric-resistant oral delivery system for improved delivery of CUR. To the best of our knowledge, exploiting KC as a major bulk phase structuring agent with gelation of CUR encapsulated in O/W emulsion is rarely reported. KC with strong gelling properties is capable of structuring and complexifying CUR-MCT into its helical form. Such a structure confers encapsulated CUR to be less susceptible to acid hydrolysis in GIT. It is hypothesized that random coiled KC chains are capable to interact with the CUR-MCT via hydrogen bonding between the KC polymeric chains and the glycerol molecules of MCT at elevated temperature. Upon cooling, KC undergoes gelation to rearrange into a more ordered, aggregated, and rod-shaped double helical conformation prior to the parallel aggregation of these double helices [37,38], which we hypothesize that this may strengthen the stability of CUR-MCT emulsion to form gel beads for GIT delivery. Meanwhile, there is so far no MCT-KC system established for the delivery of hydrophobic/lipophilic molecules. The aim of this work is also to fabricate MCT-KC gel beads to achieve a stronger gastric resistance, without the use of chemical solvents. The synthesized CUR-MCT-KC beads were solid and rigid with a spherical shape, regardless of the concentration of MCT used to dissolve the same amount of CUR molecules, as illustrated in Figure 1 below. However, CUR-MCT-KC_2 (with higher MCT concentration) beads were more yellowish in color, as seen in Figure 1b). This is due to a more even distribution and encapsulation of CUR within MCT oils with an increased volume.

#### 3.1.2. Biophysical Characterization of CUR-MCT-KC Formulation

##### SEM

**Figure 2:**
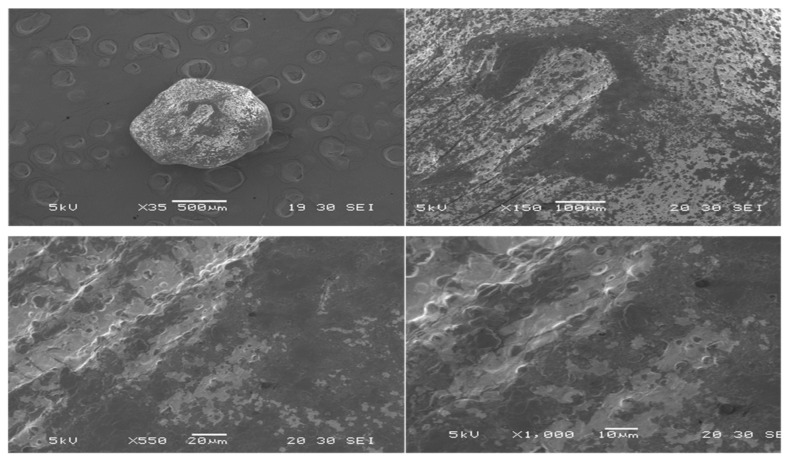
SEM analysis of CUR-MCT-KC bead showing spherical shape with moderately rough surface features. SEM analysis was performed at 5 kV over a magnification of ×35 to ×1000.

**Figure 3:**
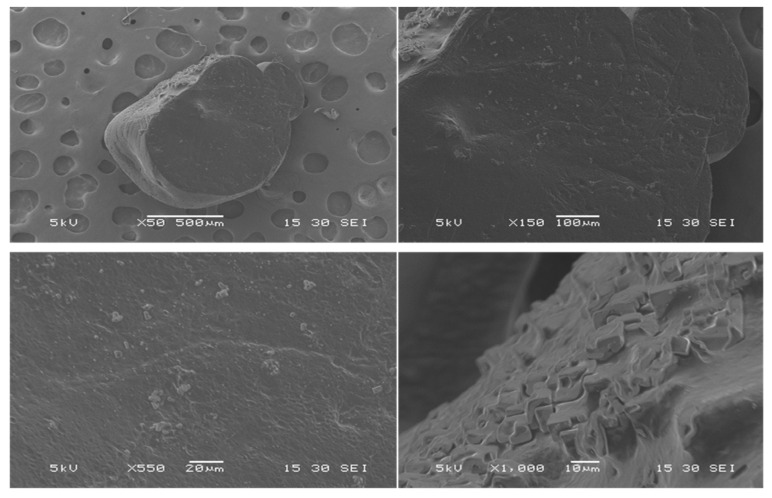
SEM analysis of CUR-MCT-KC_2 bead (higher MCT concentration used) showing smoother surface features. SEM analysis was performed at 5 kV over a magnification of ×50 to ×1000.

SEM was utilized to examine the size and surface morphology of the prepared CUR-MCT-KC formulation at different magnifications of 35×, 50×, 150×, 550×, and 1000× in Figure 2 and Figure 3. Figure 2 represents the SEM images of CUR-MCT-KC bead with a lower MCT concentration incorporated, whilst Figure 3 refers to CUR-MCT-KC_2 bead with a higher MCT concentration used to dissolve the same amount of CUR added. Particle shape, size, and surface chemistry play vital roles in the delivery and of encapsulated drugs. Both Figure 2 and Figure 3 illustrate the bead-like morphology of CUR-MCT-KC. The bead was spherical in shape with a thicker coating, a rougher surface and lesser pores, suggesting the suitability of CUR-MCT-KC in achieving a more sustained drug release pattern [39]. A rough and less porous surface promotes better encapsulation capability, bead–cell interactions, and a lower release rate [40]. Furthermore, the rough surface of CUR-MCT-KC is advantageous to ease the effective surface modifications for enhanced cell targeting capability or a desired drug release profile.

CUR-MCT-KC_2 that was comprised of a higher amount of MCT was shown to be less spherical in shape with smoother and less porous surface features, as displayed in Figure 3. This indicates that the ratio of lipid and polysaccharide composition in a complex can significantly influence the morphological characteristics of the final product. The presence of more MCT oils contribute to a smoother bead surface and gel beads tend to be more spherical in shape with increasing amounts of polysaccharides, KC acting as the structuring agent. Nonetheless, the size of CUR-MCT-KC bead was recorded as ~600 µm regardless of the MCT concentration used to dissolve the same amount of CUR added initially.

##### TGA

**Figure 4:**
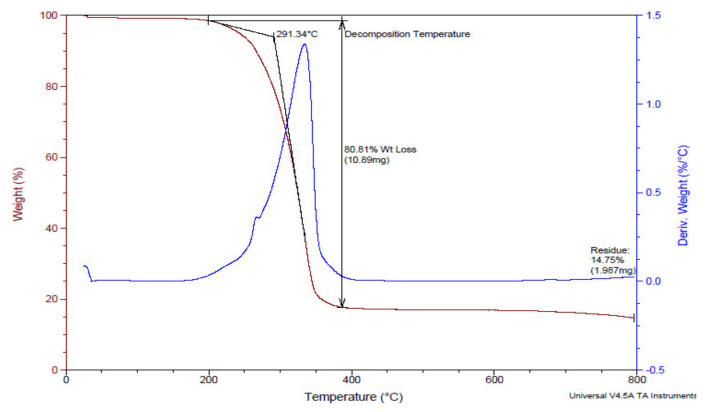
TGA analysis of CUR-MCT-KC bead to evaluate the change in weight at a constant heating rate of 10 °C/min from 30 to 600 °C. There was a sharp peak observed at approximately 320 °C with 80.81% weight loss, indicating the melting phase and decomposition of CUR-MCT-KC from 200 to 350 °C.

The thermal behavior and stability of CUR-MCT-KC was studied using the TGA characterization. Figure 4 illustrates the TGA thermograms of CUR-MCT-KC. As seen in Figure 4, the primary heat-stimulated event was observed with a small slope between 150 and 200 °C, due to a dehydration or desolvation process [41]. The maximum evaporation temperature was detected in the range of 30 °C to approximately 200 °C. There was a sharp peak observed at approximately 320 °C with 80.81% weight loss, which is related to the melting of CUR-MCT-KC, where it entered a melting phase and decomposition from 200 to 350 °C. CUR is in a crystalline form at an ambient temperature and the melting point of CUR was reported to be around 177 °C [42]. This suggests that the encapsulation of CUR in MCT-KC helps to increase its thermal stability with a higher melting point. Degradation stopped at approximately 400 °C, with a weight loss of 80.81% and a residue of 14.75%. The TGA analytical result reveals CUR-MCT-KC with good thermal stability for its various applications in the industrial process and in storage.

##### FTIR

**Figure 5:**
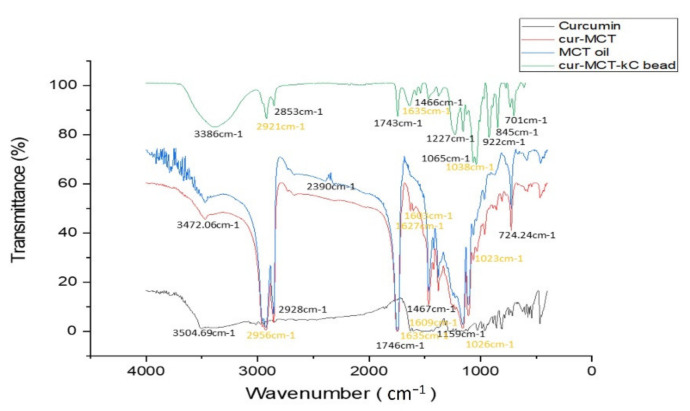
The FTIR spectra of the prepared CUR-MCT-KC, CUR, MCT oil, and KC in the range of 400–4000 cm^−1^ at a 4 cm^−1^ resolution. The yellow wavelengths indicated the successful CUR encapsulation whereby most of the active functional groups of CUR have been introduced in MCT as well as in the final formation of CUR-MCT-KC.

FTIR spectrum indices were used to differentiate between CUR, MCT, KC, and indicates the formation of CUR-MCT-KC formulation. The FTIR spectrum and vibrational characteristics of various functional groups presented in each component (CUR-MCT-KC, CUR-MCT, CUR, MCT) were identified as displayed in Figure 5 and were compared to other reported work. The FTIR bands and functional groups of pure CUR, MCT, and KC are shown in Appendix A (Table A1).

Regarding the solubility and encapsulation of CUR in MCT to form CUR-MCT, the FTIR spectra indicates most of the active functional groups of CUR have been introduced in MCT. This includes 3504 cm^−1^, 1603 cm^−1^, 1627 cm^−1^, 1023 cm^−1^, and 724 cm^−1^ attributed to the phenolic (OH), carbonyl C=O and alkenes C=C, benzene ring, C-O-C stretching vibrations, and CH_2_ stretching vibrations of CUR, respectively. On the other hand, the chemical interactions and the formation of CUR-MCT-KC were confirmed, as shown in Figure 5 with the presence of major peaks of CUR and MCT in KC such as the 3386 cm^−1^ peak with slight spectral changes due to interactions of CUR and MCT. On top of that, other functional groups associated with 1635 cm^−1^, 1605 cm^−1^, and 1038 cm^−1^ peaks showed the encapsulation of CUR; 2923 cm^−1^, 1743 cm^−1^, and 1466 cm^−1^ identified the incorporation of MCT, and 922 cm^−1^, 845 cm^−1^, and 701 cm^−1^ revealed the presence of KC. Hence, the presence of CUR, MCT, and KC functional peaks in the FTIR analysis indicates the conjugated composite materials of CUR-MCT-KC beads.

#### 3.1.3. Encapsulation Efficiency (EE) of CUR-MCT-KC Formulation

EE is the percentage of CUR being entrapped successfully into the MCT-KC formulation. The CUR-MCT-KC_2 bead exhibited a significantly higher EE (73.98 ± 1.57%) than CUR-MCT-KC bead (~24.04 ± 2.17%). EE is therefore relatively dependent on the amount of MCT oil used. This also indicates that the use of MCT can improve the solubility of CUR to a greater extent. Their storage stability was also examined with CUR-MCT-KC_2 beads showing a good stability of up to at least 15 days at room temperature (EE of 69 ± 0.034%), and 30 days of storage at −20 °C (EE of 71 ± 0.018%). Hence, CUR-MCT-KC_2 beads were selected for further investigations in terms of solubility, dissolution, and drug release studies.

The surface charge and structural diversity of the encapsulation materials used can notably affect their efficiency in encapsulating and retaining CUR. Initially, CUR interacted with the fatty acid chains of MCT that aided in its solubilization. Ma, Zeng [43] suggested that MCT improves the dipole–dipole interactions between its polar groups and CUR molecules, resulting in an enhanced CUR solubility. Furthermore, MCT consists of oxygen molecules that allow the formation of hydrogen bonds with CUR on top of their effective dipole–dipole interactions [31]. Therefore, with an increase in MCT concentration, there are more dipole–dipole and hydrogen bonds formed, leading to better CUR loading and EE. The solubility of CUR in MCT is significantly higher than in other common oils. This is due to MCT oil, which possesses shorter acyl chains (C6-C10) and higher polarity as compared to other common oils (C16-C20) such as corn, soybean, olive and rapeseed oils, and LCT oils. Hence, the polarity of MCT is more suitable for interactions with CUR molecules [25], demonstrating its effectiveness as a CUR delivery carrier.

Besides this, the emulsifier plays an important role in the formation of a stable emulsion. Span 20 with an intermediate hydrophilic-lipophilic balance value of 7−9 was used in this study. It was determined to improve the CUR solubility and effectively prevent CUR crystallization, even after being stored at room temperature for months [24]. The presence of Span 20 had lowered the interfacial tension, resulting in better CUR solubility in MCT. KC was used to encapsulate those loosely surface bound CURs and act as a protective layer on top of the MCT compartment against harsh conditions such as the acidic environment of the stomach. 

The EE (73.98 ± 1.57%) of CUR-MCT-KC_2 bead is highly comparable and performs better than other reported CUR-loaded formulations including CUR-MCT nanoemulsion with 71.5% EE [24], CUR-KC complex with 73.6% EE [22], CUR-MCT organogel with ~2.6% EE [9], CUR organogel-based nanoemulsion with 9% EE [34], CUR-encapsulated caseinate/zein nanoparticles with 62% EE [44], CUR-KC film for food freshness monitoring with 3% EE [28], and CUR-KC drug carrier to treat A549 lung cancer cells with 73% EE [22]. It is important to note that most of the reported CUR-MCT nano/micro emulsions focused on improving the lipolysis and bioaccessibility of CUR. Hence, unlike our present work, the efficiency of CUR encapsulation and loading in MCT were not studied extensively in most cases. In short, CUR-MCT-KC_2 formulation possesses better encapsulation efficiency than other existing CUR carriers, confirming its suitability to carry the hydrophobic CUR with increased solubility and stability.

### 3.2. Interactions of Hydrophobic CUR with MCT Oil

#### 3.2.1. Solubility of CUR in MCT Oil

The solubility of CUR in MCT oil was examined in this study to understand the loading capacity of CUR per ml of MCT used. The result reveals that the solubility of CUR in MCT was 1.32 ± 0.03 mg/mL, which is more than 100 times higher than the solubility of unformulated CUR in PBS (4.15 µg/mL) [45]. This can be ascribed to the shorter acyl chains and the presence of a higher number of polar groups (oxygen) of MCT that enhance the dipole–dipole interactions between CUR molecules and MCT. The obtained result is also the same or better than other reported CUR-MCT formulations, including CUR nano-emulsion with a solubility of 0.25 mg/mL [23], 1.85 mg/g [25], 0.79 ± 0.2 wt.% [33], and 2.9 mg/g [32] in MCT oil. This validates the efficiency of MCT as an oil carrier for CUR encapsulation.

#### 3.2.2. The Effect of Heat on the Solubility of CUR in MCT

**Figure 6:**
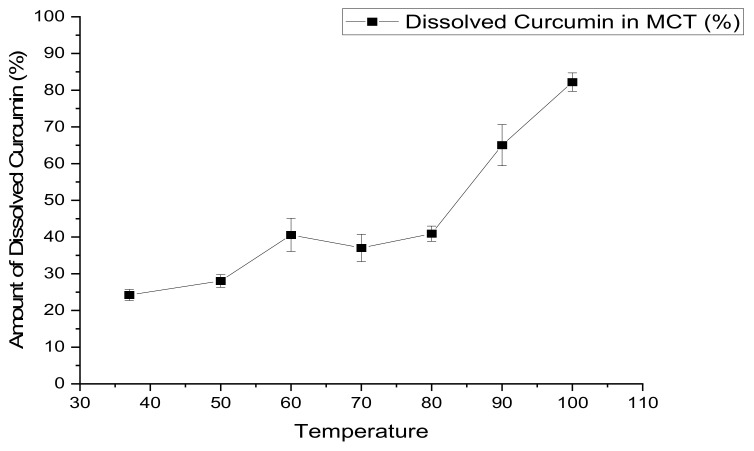
The amount of dissolved CUR (%) in heated MCT oil over a range of temperature from 37 to 100 °C in a water bath. The application of heat is suggested to improve the CUR solubility and stability in encapsulation.

CUR molecules were readily dispersed in MCT oil with increased heat, as seen in Appendix A (Figure A3). The formation and stability of CUR-MCT was investigated based on the maximum absorption peak at 425 nm using UV-visible spectrophotometry. The prepared CUR-MCT emulsion exhibited a shoulder peak at 425 nm due to the π–π type excitation of the CUR aromatic system, indicating the presence and stability of CUR in MCT oil. Changes in the absorbance intensity at 425 nm were then applied to evaluate the stability of CUR-MCT emulsion under the effect of heat when the temperature increased substantially over time. Figure 6 illustrates that the absorbance intensity increased gradually over the incubation period with increasing temperature, revealing that the solubility of CUR in MCT was enhanced under the influence of heat. Hence, CUR was able to be dissolved in heated MCT oil without degrading and remained soluble for a relatively long period of time with a stable CUR-MCT-KC bead formed, demonstrating good stability. This was proven with the 71% EE of stored CUR-MCT-KC bead at −20 °C after one month of storage time, which was close to the EE (73.98 ± 1.57%) measured right after the fabrication process. This result is aligned with other research studies, which applied heat to improve the encapsulation of CUR in MCT [9,26,33,34]. CUR formulations would have a higher retention rate and stability if they were stored at lower temperatures, in order to prevent degradation by Ostwald ripening [46]. In addition, many reported studies exemplified the high stability of CUR in MCT after a 30-day storage period at different temperatures, including room temperature at 4 °C and −20 °C [23]. Therefore, the application of heat is suggested to improve the CUR solubility and stability for an efficient CUR encapsulation.

#### 3.2.3. In Vitro Dissolution Study of CUR-MCT Formulation

**Figure 7:**
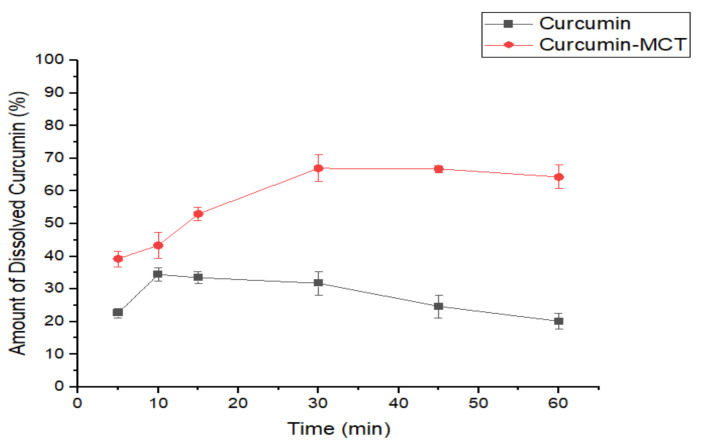
The in vitro dissolution test of unformulated CUR and CUR-MCT was conducted in PBS over 60 min at temperature 37 °C with gentle shaking of 150 rpm in a shaking incubator. MCT plays an important role in enhancing the CUR solubility.

CUR is not soluble in the water phase and its solubility is extremely low, even with the application of emulsifiers. Dissolution is defined as the rate of solute dissolving in a solution where it is a kinetic process and is measured by its rate. A simple in vitro experiment was carried out to determine the amount of CUR dissolved in PBS after an incubation period of 1 h, with/without the presence of MCT. PBS was used in the study to mimic the human physiological environment. Unlike the unformulated or naked CUR, MCT-solubilized CUR was shown with a greater in vitro dissolution rate in PBS. The data, as seen in Figure 7, suggests that CUR-MCT has a higher dissolution rate as compared to unformulated CUR alone when incubated in PBS (pH 7.4) at 37 °C, 150 rpm for an hour. The results determined that approximately 33% unformulated CUR and ~67% CUR-MCT were dissolved in PBS, respectively.

The unformulated CUR in PBS started to degrade gradually and only around 22% was detected after 1 h of incubation as compared to its initial amount added. The poor solubility of unformulated CUR (~33%) is in keeping with other studies, which revealed the high hydrophobicity and poor solubility of unformulated CUR [47,48]. This is due to the low water solubility of <0.005 wt.% and the high oil–water partition coefficient (logP 3.1) of CUR. There are also studies [49,50,51] that have demonstrated that most of the CUR (more than 90%) is degraded rapidly within 30 min of incubation in PBS and this is similar to our findings, as shown in Figure 7, that the amount of solubilized CUR decreased drastically after 15 min in PBS (~pH 7.4). In contrast, the stability of CUR increased when it was loaded in MCT, whereby CUR encapsulation inside the oil globule helps to minimize the contact of CUR with the external PBS environment. Figure 7 reflects that the presence of MCT can enhance the solubility of CUR in PBS, at which ~65% CUR-MCT was still detectable after an hour of incubation, suggesting that the solubility of CUR-MCT in PBS was increased two-fold. This indicates the significant role of MCT compartment in MCT-KC gel beads for the encapsulation of hydrophobic/lipophilic molecules. 

### 3.3. In Vitro Release Study of CUR-MCT-KC Formulation in Simulated GI Conditions

**Figure 8:**
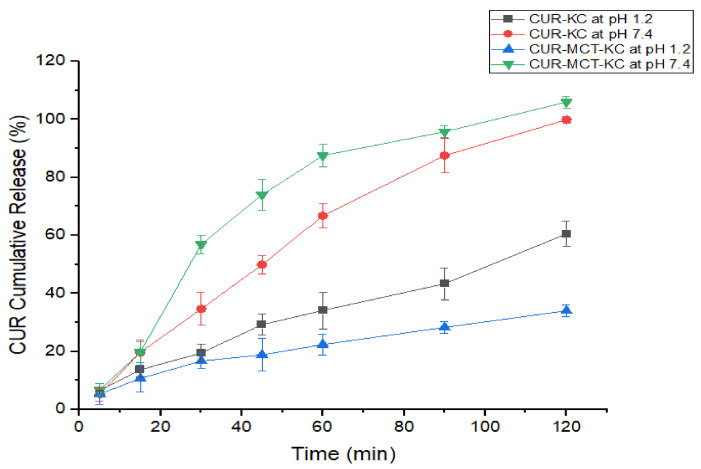
Cumulative release characteristics of CUR from CUR-KC and CUR-MCT-KC under the influence of different pH: acidic pH 1.2 and alkali pH 7.4. The release experiment was the performance at 37 °C in a PBS buffering system, with gentle shaking at 150 rpm over 2 hr. CUR release (%) was monitored at specific intervals: 5th, 15th, 30th, 45th, 60th, 90th, 120th min.

The digestion of delivery carrier in the GIT is a complex mechanism and its impact on the release of encapsulated bioactive compounds plays an important role in the uptake, distribution, and bioavailability of the encapsulated compound. Both MCT and KC were evaluated for their entrapment efficiency for CUR via the in vitro release study under human physiological conditions. This study is important to demonstrate the synergetic effects of both MCT and KC as an oral delivery carrier. The conditions of an incubating medium could crucially affect the drug release profile. Hence, both the gastric and intestinal digest were investigated in vitro for the cumulative release of CUR from MCT-KC beads, as seen in Figure 8 and Figure A4 (Appendix A).

#### 3.3.1. CUR-KC vs. CUR-MCT-KC at Different pH

The optimal hydrolysis of KC is largely dependent on the pH of the reaction mixture. As displayed in Figure 8, the CUR release rate was higher at pH 7.4 in the intestinal environment for both CUR-KC and CUR-MCT-KC, than at pH 1.2 in the gastric condition. This can be explained due to KC hydrolysis, which is optimum at a varying pH above pH 7, such as pH 7.5 [52] and pH 7.7 [53], and in the order of pH 7.4 > 0.1 M HCl > distilled water [54]. These are consistent with our findings that the KC degradation rate was higher at a neutral pH (~pH 7.4) with a rapid release of CUR molecules under the intestinal environment.

In this study, KCl was demonstrated as an effective crosslinker whereby KC became more resistant to acid hydrolysis in the stomach upon the crosslinking with K^+^ ions [55]. The presence of certain cations such as sodium (Na^+^), calcium (Ca^+^), or K^+^ ions strongly enhances the stability of the ordered helices and aggregations, which further promotes gelation, resulting in a slower depolymerization rate of KC in acidic condition. This describes the effectiveness of fabricating MCT-KC via a KCl crosslinking process in this study to obtain a more solidified carrier for hydrophobic molecules with better protective outcomes. Based on Figure 8 and Figure A4 (Appendix A), the suitability of KC in oral drug delivery was exemplified with its capability to prevent premature release and degradation of an encapsulated drug [22]. There was no initial burst at the first 15 min and less than 35% CUR was released after 1 h gastric incubation for both CUR-KC and CUR-MCT-KC beads. This elucidates the selection of KC as one of the carrier materials in this work. Importantly, CUR-MCT-KC exhibited a much lower CUR release (<30%) at the end of 2 h gastric incubation as compared to CUR-KC, suggesting that the presence of MCT further improves the acid resistance.

#### 3.3.2. In Vitro Release Profile of CUR-MCT-KC

##### In SGF

**Figure 9:**
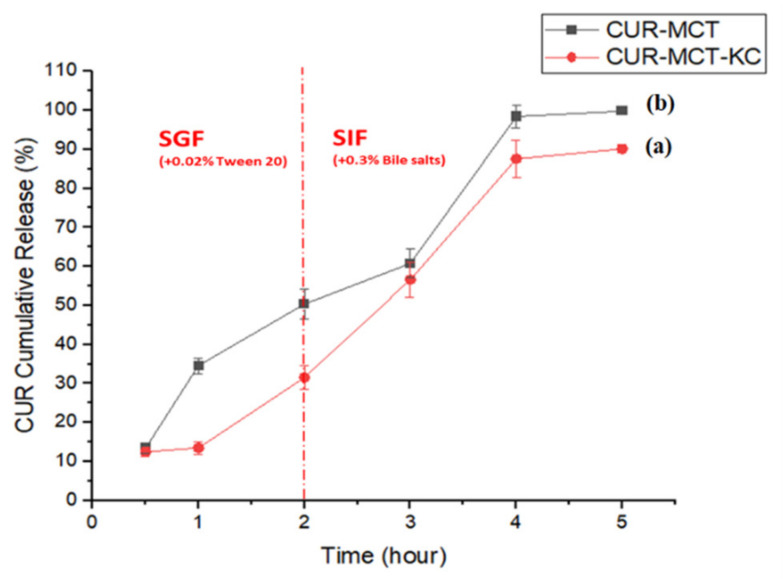
Cumulative in vitro drug release profile of CUR from (**a**) CUR-loaded-MCT-KC beads and (**b**) CUR-MCT emulsions at 37 °C with gentle shaking, 150 rpm in SGF (pH 1.2) for 2 h incubation and followed by the subsequent 3 h incubation in SIF (pH 6.8).

**Figure 10:**
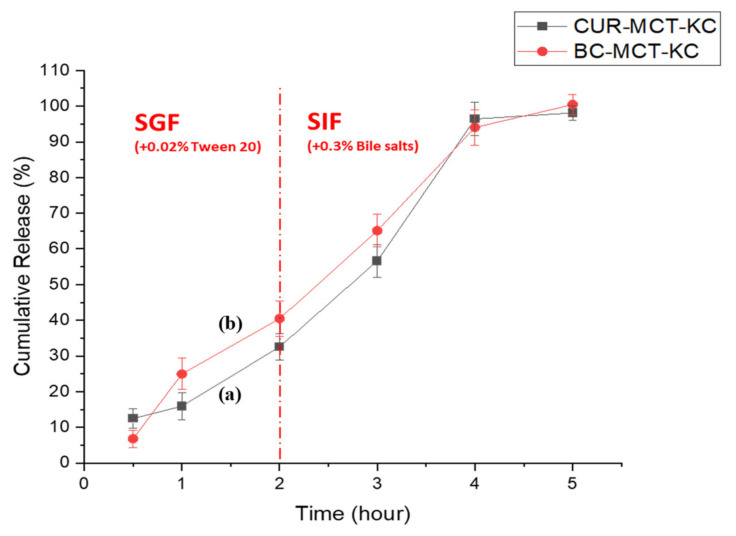
The in vitro release kinetics of MCT-KC bead in carrying two different types of hydrophobic molecules: (**a**) CUR, (**b**) BC. The cumulative release rate was investigated in simulated gastric (pH 1.2) and intestinal digestion (pH 6.8) for a 5 h incubation period, at 37 °C with gentle shaking at 150 rpm. Both curcumin-MCT-kC and beta carotene-MCT-kC beads showed similar release kinetics. There was no initial burst at the first hour and a significant release was observed at the second (<40% cumulative release) hour and an approximately 100% cumulative release was achieved at the fourth hour of incubation. Data are expressed as the mean ± standard error of three independent experiments.

**Figure A1:**
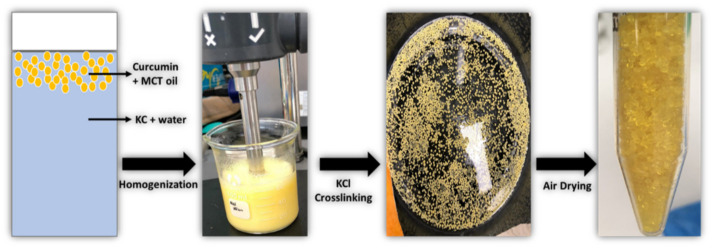
The preparation of CUR loaded-MCT encapsulated-KC beads.

**Figure A2:**
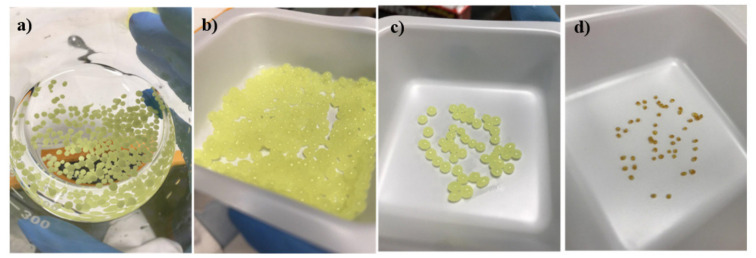
The KCl crosslinking and drying process of CUR-MCT-KC beads. (**a**) 5% KCl was used to crosslink and solidify the CUR-MCT-KC beads, (**b**) the drying process of CUR-MCT-KC beads at room temperature in a light-protected container, (**c**) beads after 1-day of drying, (**d**) dried and solidified CUR-MCT-KC beads.

**Figure A3:**
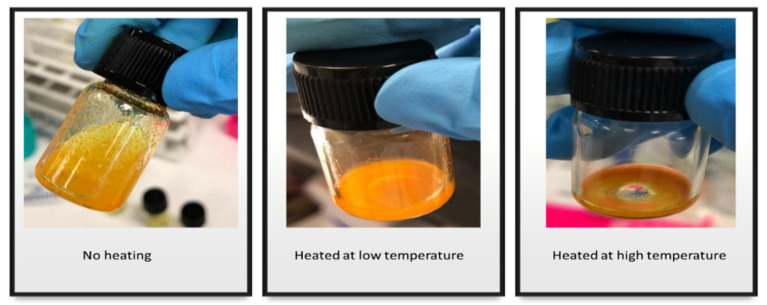
Effect of heat on the solubility of CUR in MCT. The CUR solubility escalates gradually with increasing temperature.

**Figure A4:**
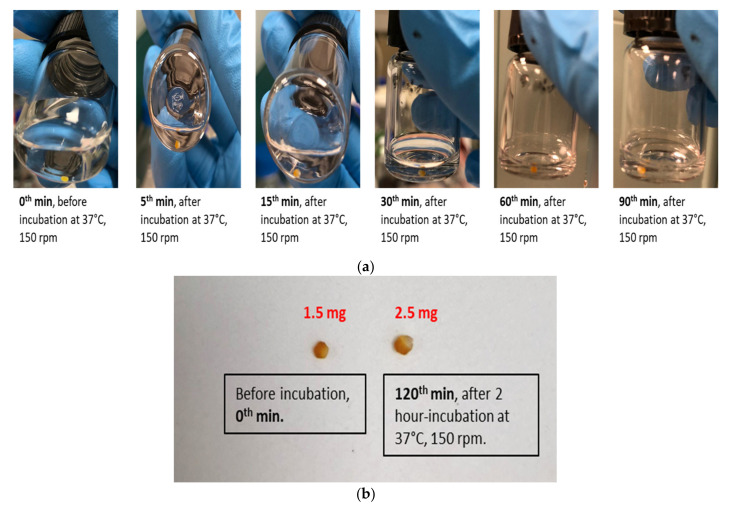
(**a**) Physical observation of CUR-MCT-KC bead at specific time intervals: 0, 5, 15, 30, 60, 90th min under the influence of acidic pH 1.2 over an incubation period of 120 min at 37°C and 150 rpm, (**b**) The different in weight of CUR-MCT-KC bead before and after the incubation (120 min) at pH 1.2 at 37°C and 150 rpm.

In the initial phase, the release (%) of CUR from CUR-MCT-KC was gradual and there was no initial burst observed at the first hour of incubation in SGF at 37 °C for 2 h, indicating that CUR-MCT-KC was relatively resistant to pepsin digestion. This ensures that the encapsulated CUR is delivered and released in the targeted intestinal region. When incubated in SIF for the following 3 h, CUR-MCT-KC was capable of releasing the maximum amount (>90%) of entrapped CUR between the third and the fourth hour of incubation in SIF, as seen in Figure 9 and Figure 10. This illustrates an efficient release of CUR under mimicked intestinal conditions, allowing further CUR absorption into the human circulatory system. A delivery system with an efficient release at the targeted site is essential for CUR to be able to elicit its bioactivity.

The release pattern of CUR-MCT-KC has specified its suitability for oral administration of drugs. Oral delivery is one of the most preferred routes, due to its high patient compliance. As shown in Figure 9, the slow release of CUR in the first 2 h has proved the resistance of MCT-KC against the harsh gastrointestinal environment of the stomach in order to avoid burst release and elicit better shielding effects for the encapsulant in the localized environment. Moreover, carrageenan could maintain its ionization at a low pH owing to their low pKa value, suggesting their potencies as gastric floating tablets [56]. In this study, the addition of monovalent cations such as KCl has resulted in a stronger KC gel [57] due to the ion pairs formed between KC and added KCl crosslinker. As a result, CUR-MCT-encapsulated KC forms a mesh or network-like layout to enhance the CUR retention and protective effects to a certain degree. This is an important feature of KC in providing an effective encapsulation when KC helices interact with CUR-MCT molecules to form a complex structure, leading to a higher solubility. The solubility can be enhanced by 15 to 30 times as compared to the free compounds due to their conformational changes into an amorphous setting in the complex structure [58]. On top of that, KC is employed in this work as it is often used as an excipient for bead fabrication due to its viscoelastic, easy gelling, and thermo reversible properties for a prolonged retention and controlled drug release [59]. In short, MCT-KC may act as a potential carrier to deliver and release hydrophobic/lipophilic drug molecules, specifically at intestinal regions.

##### CUR-MCT Emulsion vs. CUR-MCT-KC Beads

The release profile of the CUR-MCT-KC bead was compared to that of the CUR-MCT emulsion, as illustrated in Figure 9. The CUR release of the CUR-MCT emulsion was more than 50% after the 2 h gastric incubation as compared to CUR-MCT-KC beads with ~30% CUR release under the simulated gastric condition. This validated the efficiency of KC as an effective structuring agent for the encapsulation and gelation of CUR-MCT emulsions. The release profile of the CUR-MCT emulsion and the CUR-MCT-KC bead are useful as references to investigate the release pattern of MCT encapsulation, which is scarcely reported. Most reported works [1,9,23,32,33,34,35] focus on the bio-accessibility and lipolysis of MCT encapsulation in the intestinal region. However, these reported studies do not provide an in-depth understanding regarding the effectiveness of its GIT delivery in terms of encapsulation efficiency and gastric resistance of MCT as a drug carrier towards intestinal absorption. Some studies have supported the efficiencies of KC encapsulation, which includes the use of KC in delivering probiotic bacteria such as *Lactobacillus plantarum* and *Lactobacillus rhamnosus*, as well as other poorly soluble drugs for enhanced GIT delivery [22,60,61,62]. The release of CUR in SGF can be attributed to the normal swelling and hydrolysis of KC and thus contributed to the increasing release of CUR detected at the 2nd h of SGF incubation. This outcome is highly desired, because the degradation of KC can further expose the CUR-MCT compartment to SIF to ease the digestion of MCT and increase the release of CUR within the intestinal lumen.

##### Stomach pH of Fasted- and Fed-Stated

In this study, the pH used to mimic the gastrointestinal condition was extreme, at around pH 1.2. This was to investigate the protective effect of KC under the most unfavorable and rapid acidification of gastric digestion. In fact, under the in vivo condition or the human physiological environment, the gastric of GIT is acidified gradually, whilst gastric emptying is initiated as soon as food ingestion takes place. The gastric pH of a fasted- and fed-stated is ~pH 1.3 and pH 5, respectively in healthy subjects due to the buffering effects of ingested meals [63,64]. Hence, the result recommends that KC can even offer a better protective effect against the acidic pH during the fed-state and gastric emptying process.

##### In SIF

Pancreatic enzymes, also known as pancreatin, are commercial mixtures of lipase, protease, and amylase enzymes [65]. When CUR-MCT-KC was transferred to SIF, the CUR release was increased drastically between the second and the fourth incubation hour and with approximately a 100% release after 5 h of incubation, as displayed in Figure 9 and Figure 10. This can be explained with the rapid hydrolysis of MCT oils by pancreatic lipase into glycerides and free fatty acids to further release more encapsulated CUR molecules into the medium. KC belongs to a class of polysaccharides that can be degraded easily by enzymatic hydrolytic reactions, leading to the breakdown of α-1,3 and β-1,4 glycosidic linkage and the formation of galactose and oligosaccharides. However, the pancreatic amylase cleaves α-1,4-glycosidic linkages, but not the α-1,3 and β-1,4-glycosidic linkages found in KC. Therefore, KC is not degraded to harmful poligeenan and remained unaltered via the GIT. Nevertheless, α-amylase has been reported to have effects on the hydrolysis of KC up to a certain degree, which explained the higher CUR release rate in SIF [65].

The swelling ability of CUR-MCT-KC beads were larger in pH 6.8 compared to pH 1.2, due to the changes in the ionic structure of KC when exposed to a different medium. It is believed that at pH 1.2, the hydrogen bonds between CUR and KC molecules are much stronger due to the existence of a carboxylic group (COOH) of polymers and OH groups which limit swelling whilst electrostatic repulsions are intensified between ionized groups (carboxylates, COO-) at pH 6.8 of intestinal fluid, leading to greater swelling effects. Consequently, media diffusion into the beads is higher and resulting to a higher release of encapsulated CUR in SIF [59].

In SIF, the presented bile salts play a role in changing the interface, which facilitates the lipase-based degradation and thus enhances CUR release. It was reported that MCT is highly favorable over LCT, because MCT can be directly absorbed via the portal vein to the liver without the formation of chylomicrons [31]. This makes them highly preferable especially for patients with bile salt or pancreatic lipase deficiency. LCT, on the other hand, requires a more complex absorption process whereby they are modified into chylomicrons and transported via the thoracic duct lymph system to the liver for metabolism [10]. Furthermore, a shorter MCT is highly desirable due to its more rapid lipolysis to release more CUR in a short time for faster absorptions, as compared to LCT, suggesting that this couple improves the bio-accessibility of CUR to a greater extent [66]. This explains the selection of MCT as an oil phase/carrier over other types of oil in this study.

It is proposed that released CUR from hydrolyzed MCT conjugates can then diffuse through enterocytes more rapidly to interact with other lipid moieties to achieve a higher lymphatic drug transport. This is more effective than CUR-LCT conjugates. Theoretically, there are two possible mechanisms which occur to release the CUR molecules upon MCT lipolysis. MCT lipolysis releases a higher amount of CUR within a short time. Released CUR molecules attach and diffuse passively across enterocytes with a greater uptake efficiency in a shorter time due to its small size. Hydrophobic CUR molecules can then be solubilized with other fatty acids or monoglycerides into chylomicrons to be diffused into the lipid absorption pathways towards systemic blood circulation via the intestinal lymphatic transport, which bypassed the first-pass metabolism in the liver [66]. This reduces the CUR inactivation in liver and increases the amount of CUR available at cellular sites and thus enhances the drug bioavailability. Further studies will be conducted in the future to investigate the lipolysis rate and mechanism of CUR-MCT-KC gel beads.

There was no initial burst at the first hour for CUR-MCT-KC formulation. Although the burst release of CUR-MCT was higher (approximately 35%), MCT oil is still capable of providing some shielding effect to the encapsulated CUR as compared to naked CUR molecules, which are susceptible to gastric digestion. A higher release was observed at the second hour from the CUR-MCT emulsion (>50% cumulative release) as compared to CUR-MCT-KC beads (<30% cumulative release), indicating the shielding effect of KC under an extreme acidic environment. Data are expressed as the mean ± standard error of three independent experiments.

#### 3.3.3. In Vitro Release Profile of Beta Carotene (BC)-Loaded MCT-KC Formulation

To achieve a comparable encapsulation and desired GIT drug release pattern, MCT-KC delivery system was also used to encapsulate another drug model, BC to examine the potency of MCT-KC formulation in delivering different hydrophobic molecules. A similar release trend was observed in Figure 10, when MCT-KC was employed to encapsulate BC. This indicates a constant performance of MCT-KC in delivering poorly soluble compounds via the GIT. The BC-loaded MCT-KC was revealed to be relatively stable to the gastric enzymes but can be degraded with subsequent BC release in the presence of pancreatin and increasing pH under simulated intestinal condition.

## 4. Conclusions

The ultimate use of natural polymers and food-grade composites has become an attractive approach in current pharmaceutical delivery applications, attributed to their biological origin, biodegradability, and non-toxicity. In this study, CUR was formulated into MCT-KC to improve its solubility, encapsulation, and acid resistance. FTIR spectra and SEM images have confirmed the formation of the CUR-MCT-KC formulation. MCT-KC has also demonstrated its capability to efficiently encapsulate and release CUR under the simulated upper tract GI conditions. KC was shown with improved acid resistance at extreme pH 1.2 and simulated gastric condition, indicating its potency in offering an additional shielding effect to the encapsulated CUR. The solubility of CUR was improved using MCT two-fold with an encapsulation efficiency of 73.98 ± 1.57% and a loading capacity of 1.32 ± 0.03 mg CUR/mL MCT, suggesting the suitability of MCT as a carrier for hydrophobic compounds. The release profile of MCT-KC has revealed the delivery system with no premature burst of CUR and suppressed release under extreme simulated gastric condition (<35% release), followed by a drastic release of CUR in the mimicked intestinal condition (~100% release after 3 h incubation), which is favorable for further CUR absorption into the systemic circulation. CUR-MCT-KC beads can be stabled for at least one month at −20 °C storage conditions. On the whole, findings from this work has demonstrated CUR loaded MCT-encapsulated KC formulation which could be potential materials for the development of natural, food-graded and polymer-derived delivery vehicles with enhanced characteristics for pharmaceutical applications. Further in vivo research will be carried out to explore more valuable functions and the potential of CUR-MCT-KC as an oral delivery system.

## Data Availability

Not applicable.

## Appendix A

**Table A1:** The FTIR absorption bands and associated functional groups of individual CUR, MCT, and KC.

Component	FTIR Absorption Band	Descriptions—Characteristics of Different Functional Group	References
CUR	3504.69 cm^−1^	Assigned to the stretching vibrations of phenolic hydroxyl (O–H) group	[1,47]
	1628 cm^−1^	Related to the overlapping stretching vibrations of carbonyl C=O and alkenes C=C vibrations	
	1605 cm^−1^	Indicated the stretching vibrations of benzene ring	
	1026/856 cm^−1^	Attributed to the C–O–C stretching vibrations	
	724.24 cm^−1^	Due to CH_2_ stretching vibrations of alkene group	
MCT	3472.06 cm^−1^	Ascribed to the C=O of ester	
	2928 cm^−1^	Due to the CH_2_ and CH_3_ vibration. (asymmetric stretching vibrations of C–H of aliphatic CH_2_ group; asymmetric stretching vibration of CH of aliphatic CH_3_ groups, which attributed to the alkyl of triglycerides found in large amounts in vegetable oils)	
	1746.89 cm^−1^	Attributed to the C=O stretching vibration (ester carbonyl functional groups of the triglycerides, C=O)	
	1467.49 cm^−1^	Indicated the stretching of CH_2_. (bending vibration of C–H of CH_2_ and CH_3_ aliphatic groups)	
	1159.58 cm^−1^	Assigned to C–O vibration (stretching vibration of C–O ester groups)	
KC	1223.64 cm^−1^, 1243.07 cm^−1^	Assigned to the S=O of sulfate esters	
	1035.17 cm^−1^, 1035.62 cm^−1^	Ascribed to the glycosidic linkage, C–O–C of 3,6 anhydro-D-galactose	
	921.94 cm^−1^, 921.04 cm^−1^	Due to C–O of 3,6-anhydro-D-galactose	
	845.61 cm^−1^, 844.76 cm^−1^	Attributed to the C–O–SO_3_ of D-galactose-4-sulfate	

A simple experiment was also conducted to observe how CUR-MCT-KC bead behaves when incubated under acidic environment, at 37°C, pH 1.2, and 150 rpm for 2 h. This experiment is essential to confirm the CUR release profile and determine if KC layer capable of shielding the encapsulated CUR-MCT against gastric pH. Based on the physical observation, CUR-MCT-KC particle was stable in acidic pH with reasonable swelling as seen in Figure A4. The swelling was due to hydrogel characteristics of KC, resulting in increased weight from 1.5 mg to 2.5 mg after 2-h incubation at pH 1.2. There was no yellowish color observed in the incubated medium and the amount of released CUR was determined based on the in vitro release study in both SGF and pH 1.2.
